# Protective Mechanism of Broad Bean Extract on Parkinson’s Disease Model Cells

**DOI:** 10.3390/foods14183244

**Published:** 2025-09-18

**Authors:** Xuhao Chen, Qiang Gao, Tingting Li, Jiajia Zhao, Yujiao Liu, Xuejun Wang, Mingcong Fan, Haifeng Qian, Yan Li, Li Wang

**Affiliations:** 1 State Key Laboratory of Food Science and Technology, National Engineering Research Center for Functional Food, School of Food Science and Technology, Jiangnan University, 1800 Lihu Avenue, Wuxi 214122, China; 18605107938@163.com (X.C.); qiang1_gao@163.com (Q.G.); fanmingcong@yeah.net (M.F.); qianhaifeng@jiangnan.edu.cn (H.Q.); liyan0520@jiangnan.edu.cn (Y.L.); 2 Department of Food Science and Engineering, College of Light Industry and Food Engineering, Nanjing Forestry University, Nanjing 210037, China; litingting@njfu.edu.cn; 3 College of Cooking Science and Technology, Jiangsu College of Tourism, 88 Yuxiu Road, Yangzhou 225000, China; zjj221906@163.com; 4 College of Agriculture and Forestry, Qinghai University, 251 Ningda Road, Xining 810016, China; 13997058356@qhu.edu.cn; 5 Nantong Agricultural Science Research Institute, 28 Xingfu Road, Nantong 226012, China; wangxj4002@sina.com

**Keywords:** broad bean, Parkinson’s disease, levodopa, 6-gingerol, synergistic effect

## Abstract

Broad beans, natural sources of L-DOPA and bioactive phenolics show promise for Parkinson’s disease intervention. This study investigated broad bean extracts’ protective mechanisms against PD pathogenesis. Among screened varieties, QC25 extract exhibited optimal protection in MPP^+^-injured PC12 cells, improving viability, reducing LDH release, and mitigating cell cycle arrest. QC25 extract rescued mitochondrial dysfunction by suppressing ROS, restoring membrane potential, normalizing Ca^2+^ homeostasis, and recovering ATP synthesis. Metabolomics identified glycerophospholipid metabolism as the core protective pathway, mediating mitochondrial membrane stabilization. QC25 extract further activated PINK1/Parkin-mediated mitophagy, upregulating PINK1 and Parkin expression. Crucially, 6-gingerol—uniquely detected in QC25 extract—synergized with L-DOPA, enhancing cell viability and amplifying mitophagy through complementary mitochondrial repair mechanisms. These findings demonstrate QC25 broad bean variety exerts’ protective effects on PD model cells by regulating mitochondrial function and mitophagy, and its unique component 6-gingerol synergizes with L-DOPA to strengthen these effects. This study provides a theoretical basis for the development of QC25 as a functional food ingredient for neurological health maintenance.

## 1. Introduction

Parkinson’s disease (PD) affects over 10 million individuals globally, posing a dual therapeutic challenge: alleviating dopamine deficiency while addressing mitochondrial dysfunction—the core pathological mechanism driving neurodegeneration in 90% of idiopathic PD cases [1,2]. Although synthetic levodopa (L-DA) remains the primary therapy, its limitations—failure to suppress oxidative stress, restore mitochondrial homeostasis, or halt disease progression—highlight the shortcomings of single-target pharmacotherapy [3,4]. Mitochondrial abnormalities, marked by excessive reactive oxygen species (ROS) production, Adenosine Triphosphate (ATP) depletion, and defective mitophagy, are recognized as upstream triggers of dopaminergic neuron loss [5,6]. This mechanistic complexity necessitates multi-target interventions, underscoring the potential value of functional foods as a source of synergistic neuroprotective compounds.

In preclinical PD research, cellular models play a critical role in elucidating pathogenic mechanisms and evaluating therapeutic candidates. Among these, MPP^+^ (1-methyl-4-phenylpyridinium)-induced neurotoxicity models are widely adopted due to their ability to recapitulate key pathological features of PD [7]. MPP^+^, a metabolite of the neurotoxin MPTP (1-methyl-4-phenyl-1,2,3,6-tetrahydropyridine), selectively accumulates in dopaminergic neurons via the dopamine transporter and inhibits mitochondrial complex I activity—mimicking the mitochondrial dysfunction observed in PD patients [8]. This inhibition triggers a cascade of events, including ROS overproduction, mitochondrial membrane potential collapse, ATP depletion, and ultimately dopaminergic cell death, making MPP^+^-treated cells a validated model for studying PD-related neurodegeneration and screening neuroprotective agents [9,10].

The rise in dietary neuroprotection offers new avenues for PD management. Epidemiological studies link polyphenol-rich diets to reduced PD risk, attributed to their antioxidant and anti-inflammatory properties [11,12,13]. However, few foods naturally combine neurotransmitter precursors with mitochondrion-stabilizing phytochemicals. Broad beans (*Vicia faba* L.), an ancient crop cultivated since the Neolithic era and consumed worldwide, uniquely bridge this gap [14]. As a rich natural source of L-DA, broad beans directly replenish dopamine [15]. More importantly, their value extends beyond L-DA: preliminary studies suggest synergistic interactions between L-DA and co-occurring phenolics in mitigating oxidative damage and enhancing mitochondrial resilience [16]. However, the mechanisms underlying this phytochemical synergy remain unelucidated, and cultivar-specific variations in bioactivity have long been overlooked.

In this study, the neuroprotective effects of extracts from ten broad bean varieties on Parkinson’s disease model cells were evaluated under the condition of identical levodopa concentrations. The optimal variety was identified through a comprehensive assessment of bioactive components, cell viability, and mitochondrial protection. Subsequently, to explore the reason for the superior efficacy of this variety, we conducted a component analysis of its extract and discovered a specific compound that may enhance the effect of levodopa. Validation experiments, which assessed cell viability, mitochondrial function, and the mitophagy pathway, confirmed that this compound exerts a synergistic neuroprotective effect with levodopa. This research indicates that broad beans provide a basis for the development of natural neuroprotective foods. The bioactive components in broad beans can act as potentiators, improving the efficacy of levodopa while alleviating oxidative stress. These findings offer new directions for key formulations in the design of functional foods for neurological health.

## 2. Materials and Methods

### 2.1. Materials and Reagents

Ten broad bean varieties (QC25:*Vicia faba* L. var. *equina*, 4030:*Vicia faba* L. var. minor, rf94:*Vicia faba* L. var. *major*, QC14:*Vicia faba* L. var. *major*, QC15:*Vicia faba* L. var. *major*, QC16:*Vicia faba* L. var. *major*, QC18:*Vicia faba* L. var. *major*, QC19:*Vicia faba* L. var. *equina*, QC21:*Vicia faba* L. var. *major*, lf18:*Vicia faba* L. var. *equina*) were obtained from the Nantong Institute of Agricultural Sciences and the Qinghai Academy of Agriculture and Forestry Sciences. PC12 cells were purchased from the Cell Bank of the Chinese Academy of Sciences. L-DA, MPP^+^ and 6-gingerol were purchased from Yuan Ye Biotechnology Co., Ltd. (Shanghai, China). MTT was purchased from Solarbio Science & Technology Co., Ltd. (Beijing, China). Acetonitrile and formic acid (used in UHPLC-Q/TOF-MS) were purchased from Sinopharm Chemical Reagent Co., Ltd. (Shanghai, China). The CheKine™ ATP assay kit was obtained from Yeasen Biotech Co., Ltd. (Wuhan, China). LDH assay kit, ROS assay kit, Fluo-4 calcium assay kit, JC-1 mitochondrial membrane potential assay kit, cell cycle kit, cell apoptosis assay kit and qPCR kit were purchased from Beyotime Biotechnology Co., Ltd. (Shanghai, China).

### 2.2. Preparation of Broad Bean Extract

Broad bean samples were dried, ground into powder, and sieved through an 80-mesh sieve. For extraction, the powder was mixed with an extraction solvent consisting of acetonitrile, water, and formic acid (50:50:1, *v*/*v*/*v*) at a solid-to-liquid ratio of 100 g/L. Ultrasonic extraction was performed at a power of 300 W. After extraction, the mixture was centrifuged at 8000 rpm for 15 min. The supernatant was filtered, and the filtrate was concentrated and lyophilized.

### 2.3. Determination of the Components of Broad Bean Extract

#### 2.3.1. Determination of L-DA Content

L-DA content was quantified using a modified high-performance liquid chromatography (HPLC) method. The chromatographic conditions were as follows: mobile phase, acetonitrile (CH_3_CN): 1‰ formic acid (HCOOH) aqueous solution (20:80, *v*/*v*); detection wavelength, 280 nm; flow rate, 1 mL/min; column temperature, 35 °C. A standard curve was generated using L-DA standards (5–1000 μg/mL). Approximately 0.01 g of sample powder was extracted with 5 mL of 0.1 mol/L acetic acid (HAC) solution by sonication for 20 min. After filtration through a 0.22 µm filter, the sample was analyzed by HPLC.

#### 2.3.2. Determination of Total Phenolic Content

Total phenolic content was determined using the Folin–Ciocalteu method [17].

#### 2.3.3. Determination of Total Flavonoid Content

Total flavonoid content was determined using the NaNO_2_-Al(NO_3_)_3_ colorimetric method [18].

### 2.4. Effects of Broad Bean Extract on PD Model Cell

#### 2.4.1. Cell Culture

PC12 cells were cultured in Dulbecco’s Modified Eagle Medium (DMEM) medium (supplemented with 10% fetal bovine serum and penicillin/streptomycin) in a 37 °C incubator with 5% CO_2_. The medium was changed every 24 h. When the cells reached over 90% confluency, they were passaged using trypsin digestion, and logarithmically growing cells were used for subsequent experiments.

#### 2.4.2. Experimental Grouping

The experiment consisted of 12 groups: the control group (CON, normal culture without treatment), the model group (MPP^+^, treated with 500 µmol/L MPP^+^ for 24 h) [19], and experimental groups including QC25, 4030, rf94, QC14, QC15, QC16, QH18, QC19, QC21, and lf18. In the experimental groups, after MPP^+^ treatment, extracts of broad beans corresponding to 100 µg/mL L-DA content were added.

#### 2.4.3. Cell Viability Assay

Cells were seeded in 96-well plates. After 24 h drug treatment according to the groupings described in Section 2.4.2, 100 µL of 3-(4,5-Dimethylthiazol-2-yl)-2,5-Diphenyltetrazolium Bromide (MTT) solution (0.5 g/L) was added to each well and incubated at 37 °C for 4 h. Subsequently, 150 µL of Dimethyl Sulfoxide (DMSO) was added to each well to dissolve the formazan crystals. The optical density was measured at 490 nm wavelength using a microplate reader (Varioskan LUX, Thermo Fisher Scientific, Waltham, MA, USA).

#### 2.4.4. Measurement of Cell LDH Release

Cells were seeded in 96-well plates. Following 24 h drug treatment according to Section 2.4.2, with additional setup of maximum enzyme activity control wells and blank control wells, 60 µL of Lactate Dehydrogenase (LDH) detection working solution was added to each well. The absorbance at 490 nm was determined using a microplate reader, and the LDH release rate was calculated for each well.

#### 2.4.5. Measurement of Cell Cycle

Cells were cultured in 6-well plates and assigned to the following groups: model group, blank group, QC25 group, rf94 group, and QC15 group. Drug administration was performed as described previously. After incubation, cells were washed with PBS, were first fixed with 70% ethanol, and then stained with a Propidium Iodide (PI) staining working solution prepared according to the kit instructions. After removal of unbound PI, cells were loaded onto a flow cytometer and detected at an excitation wavelength of 488 nm.

### 2.5. Effects of Broad Bean Extract on Mitochondria in PD Model Cell

#### 2.5.1. Measurement of ROS Concentration

Cells were cultured to the logarithmic growth phase and seeded in plates. After 24 h treatment of the control group, model group, and QC25 group, the cells were washed with pre-cooled buffer to remove the culture medium. Subsequently, DCFH-DA was added and co-incubated with the cells for 30 min, followed by washing to remove unbound probes. Fluorescence images were captured under an inverted fluorescence microscope at high magnification, and fluorescence intensity was measured using a microplate reader (excitation wavelength: 488 nm, emission wavelength: 525 nm).

#### 2.5.2. Mitochondrial Membrane Potential Detection

Cells were seeded in 12-well plates and cultured to the logarithmic phase, followed by drug treatment according to the groupings described in Section 2.5.1. After washing with buffer, cells were incubated with JC-1 reagent diluted to an appropriate concentration at 37 °C for 30 min, followed by PBS washing. Fluorescence signals were detected using a flow cytometer, and the mitochondrial membrane potential was evaluated based on the fluorescence intensity ratio (red/green) (excitation wavelength: 490 nm, emission wavelength: 530 nm).

#### 2.5.3. ATP Content Measurement

Cells were seeded in 12-well plates and cultured to the logarithmic phase, followed by drug treatment according to Section 2.5.1. After 24 h, cell lysis buffer was added. Post-lysis, the lysate from each well was centrifuged at 12,000 rpm and 4 °C for 15 min. The supernatant was collected and mixed with the reaction solution from an ATP detection kit. After incubation at 37 °C for 20 min, fluorescence intensity at 700 nm was immediately measured using a fluorometric microplate reader. The fluorescence intensity was proportional to ATP content.

#### 2.5.4. Measurement of Ca^2+^ Concentration

Cells were seeded in 12-well plates and cultured to the logarithmic phase, followed by drug treatment according to Section 2.5.1. After thorough Phosphate-Buffered Saline (PBS) washing to remove residual medium, cells were incubated with the fluorescent probe Fluo-4 at 37 °C for 30 min, followed by buffer washing to remove unbound Fluo-4. Fluorescence intensity was detected using a microplate reader (excitation wavelength: 488 nm, emission wavelength: 520 nm).

### 2.6. UPLC-QTOF/MS Quantitative Analysis

#### 2.6.1. Sample Extraction

Cell culture and drug treatment procedures followed Section 2.5.1. After washing twice with pre-cooled PBS (4 °C), 2 mL of 80% methanol was added to quench cellular metabolism. Cells were scraped using a cell lifter and transferred to centrifuge tubes. The samples were centrifuged at 13,000 rpm for 15 min, and the supernatant was collected and dried under nitrogen gas. Metabolites were reconstituted in 200 µL of resuspension solution (acetonitrile:methanol = 7:3), followed by centrifugation at 13,000 rpm for 10 min. The supernatant was injected for analysis. Quality control (QC) samples, prepared by pooling equal volumes of metabolites from all samples, were used to evaluate experimental reproducibility.

#### 2.6.2. Instrumental Analysis

The chemical profiling of the extracts was conducted on a Waters Acquity Ultra Performance Liquid Chromatography-Quadrupole Time-of-Flight Mass Spectrometry (UPLC-QTOF/MS) system (Acquity, Waters Corporation, Milford, MA, USA). The detailed analytical parameters were as follows:

Liquid Chromatographic Conditions:

Chromatographic separation was achieved using an Acquity BEH C18 column (2.1 mm × 100 mm, 1.7 μm). The mobile phase consisted of (A) acetonitrile and (B) 0.1% aqueous formic acid. A gradient elution program was applied at a flow rate of 0.3 mL/min: 0–1 min, 5% A; 1–13 min, 5–99% A; 13–13.5 min, re-equilibration to 5% A, followed by a 3 min post-run stabilization. The injection volume was 1 μL.

QToF Mass Spectrometric Conditions:

Ionization was performed with a cone voltage of 35 V. The desolvation gas (N_2_) flow rate was set to 600 L/h, with source and desolvation temperatures maintained at 100 °C and 400 °C, respectively. Argon served as the collision gas. Data acquisition was carried out in full-scan mode (*m*/*z* 50–1200).

#### 2.6.3. Data Processing

Upon completion of detection, raw data were imported into Progenesis QI for metabolomic processing. The identification of compounds was mainly based on the METLIN database, where a score threshold of 30 or higher was applied. Key parameters including mass-to-charge ratios (*m*/*z*), ion intensities, and sample identifiers were subsequently subjected to multivariate pattern recognition analysis using SIMCA-P software (v11.0, Sweden). Principal component analysis (PCA) and partial least squares-discriminant analysis (PLS-DA) were employed to characterize metabolic profiles. Model validity was rigorously validated through cross-validation (Q^2^ > 0.5 threshold) and permutation testing (200 iterations, *p* < 0.05).

### 2.7. RT-PCR

Cell culture conditions, experimental grouping, and drug treatments were performed as described in Section 2.5.1. Total RNA was extracted according to the kit instructions, and RNA concentration was determined. Using a reverse transcription kit, mRNA was reverse transcribed into cDNA. cDNA was used as the template, and GAPDH was used as the internal control. Amplification and detection were performed using a PCR machine. The PCR amplification conditions were as follows: 95 °C for 30 s for pre-denaturation, 95 °C for 5 s for denaturation, 60 °C for 60 s for annealing, with a total of 40 cycles; the melting curve was 95 °C for 5 s, 65 °C for 60 s, followed by cooling at 50 °C for 30 s. Each experimental indicator was tested in triplicate, and the average value was taken. Data were analyzed using the 2^−ΔΔCt^ method. The primer sequences are shown in Table 1.

### 2.8. Screening for Levodopa Synergistic Substances

The three broad bean varieties (QC25, rf94, and QC15) demonstrating optimal protective effects on PD model cells were selected for compositional analysis. Extracts were characterized under standardized L-DOPA concentration conditions using modified chromatographic parameters. While the instrumental methodology followed Section 2.6.2, the liquid chromatographic gradient program was optimized as follows: 0–2 min, 0–10% A; 2–6 min, 10–40% A; 6–8 min, 40–100% A; 8–13 min, return to initial conditions. Data acquisition was performed using MassLynx 4.2 software, and subsequent processing utilized Unify Scientific Information System. The databases employed for the Unify analysis were the Waters Traditional Medicine Library and The University of Ottawa Phytochemical Library.

### 2.9. Effects of 6-Gingerol Combined with Levodopa on PD Model Cell

Prior to the formal experiment, a concentration optimization assay was conducted to determine the optimal working concentrations of L-DA and 6-gingerol. Logarithmic-phase PC12 cells were seeded in 96-well plates at a density of 5 × 10^3^ cells/well and cultured for 24 h. For L-DA screening, cells were treated with gradient concentrations of L-DA (10 μg/mL, 50 μg/mL, 100 μg/mL, 200 μg/mL) for 48 h, with untreated cells serving as the blank control and MPP^+^-injured cells as the model control. For 6-gingerol screening, cells were pre-treated with gradient concentrations of 6-gingerol (1 μm/mL, 5 μm/mL, 10 μm/mL, 20 μm/mL) for 4 h, followed by co-incubation with MPP^+^ for another 48 h. The MTT assay was used to detect cell viability, and the results confirmed that the optimal concentration of L-DA was 100 μg/mL and that of 6-gingerol was 10 μM. Subsequently, the experiment was divided into the following groups: control group (untreated), model group (treated with 500 μmol/L MPP^+^), L-DA group, 6-gingerol group, and L-DA + 6-gingerol group. All groups were treated with the optimal drug concentrations determined by the aforementioned screening assay. Experimental procedures referred to Section 2.4, and cell viability, LDH release rate, and cell cycle were measured for each group, respectively.

### 2.10. Effects of 6-Gingerol Combined with Levodopa on Mitochondria

Cells were cultured and grouped as described in previous methods. Drug treatments were administered following Section 2.10. Reactive oxygen species (ROS) release, intracellular Ca^2+^ concentration, mitochondrial membrane potential, and ATP levels were quantified across all groups.

### 2.11. Effects of 6-Gingerol Combined with Levodopa on the PINK1/Parkin Pathway

Drug treatments were administered following Section 2.10. Total RNA was extracted using the protocol outlined in Section 2.6. mRNA expression levels of PINK1, Parkin, NDP52, and OPTN were measured in each experimental group.

### 2.12. Statistical Analysis

Metabolomic data were analyzed using principal component analysis (PCA) and partial least squares-discriminant analysis (PLS-DA) with Simca-P 14.1 software (Umetrics AB, Umeå, Sweden) to investigate the cellular metabolomic changes caused by the process. Differential metabolites were identified using PLS-DA models (VIP > 1 and *p* < 0.05).

Other results are expressed as mean ± standard error of the mean. Multiple group comparisons were performed using one-way analysis of variance (ANOVA), followed by Tukey’s or Dunnett’s test. A *p* value < 0.05 was considered statistically significant. Graphics were plotted using Origin 2024 (OriginLab Corporation, Northampton, MA, USA).

## 3. Results and Discussion

### 3.1. Determination of Active Components in Broad Bean Extract

The contents of L-DA, total phenols, and total flavonoids were determined for different broad bean varieties, and the results are shown in Table 2. It can be observed that the variety rf94 had the highest L-DA content, with mass fractions of 15.24%, 14.89% for QC25, and 10.53% for QC14. Although QC25 does not have the highest L-DA content, it exhibits superior levels of both total flavonoids and total phenols compared to the other varieties.

### 3.2. Effect of Broad Bean Extract on the Cell Activity

**Figure 1:**
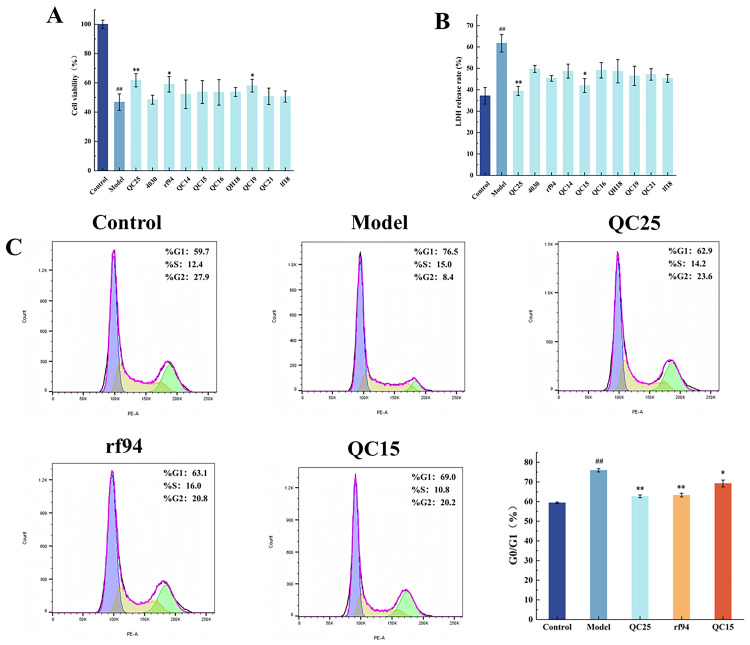
Effects of Different Broad Bean Extracts on Cell Activity in a Parkinson’s Disease Model. (**A**) Cell Viability. (**B**) Cell LDH Release Rate. (**C**) Cell Cycle. Data are shown as the mean ± SEM, *n* = 5, ^##^ *p* < 0.01 versus Control group; * *p* < 0.05, ** *p* < 0.01 versus Model group.

In this experiment, a cellular model was established using 500 µmol/L MPP^+^. MTT assay results (Figure 1A) showed that compared to the control group, cell viability in the model group was significantly reduced to 46% (^##^ *p* < 0.01). Compared to the model group, cell viability in the QC25, rf94, and QC19 groups was significantly increased, with the QC25 group achieving a cell survival rate of 61.44% (** *p* < 0.01). In the LDH release assay results (Figure 1B), the LDH release rates in the QC25 and QC15 groups were 39% and 41%, respectively, significantly lower than that of the model group. To further identify the extract offering the optimal protective effect on the Parkinson’s disease model cells, extracts from the three varieties QC25, rf94, and QC15 were selected for intervention, followed by cell cycle analysis. As shown in Figure 1C, relative to the model group, the QC25-treated group exhibited the most pronounced decrease in the proportion of cells in the G0/G1 phase (** *p* < 0.01), indicating enhanced cell division and alleviation of apoptosis. These results collectively demonstrate that the QC25 variety provides the best neuroprotective effect against damage in the PD cell model. Therefore, the QC25 variety was selected for subsequent experiments.

### 3.3. Effects of Broad Bean Extract on Mitochondrial Function

Mitochondria, as the central hubs of cellular energy metabolism, dynamically regulate a sophisticated network involving reactive oxygen species (ROS), membrane potential, calcium ion (Ca^2+^) concentration, and ATP levels [20]. During oxidative phosphorylation, the mitochondrial electron transport chain generates ROS primarily through Complexes I and III. Excessive ROS accumulation triggers mitochondrial membrane potential (ΔΨm) collapse, induces abnormal opening of the mitochondrial permeability transition pore (mPTP), and subsequently leads to cytosolic Ca^2+^ overload and impaired ATP synthesis [21].

**Figure 2:**
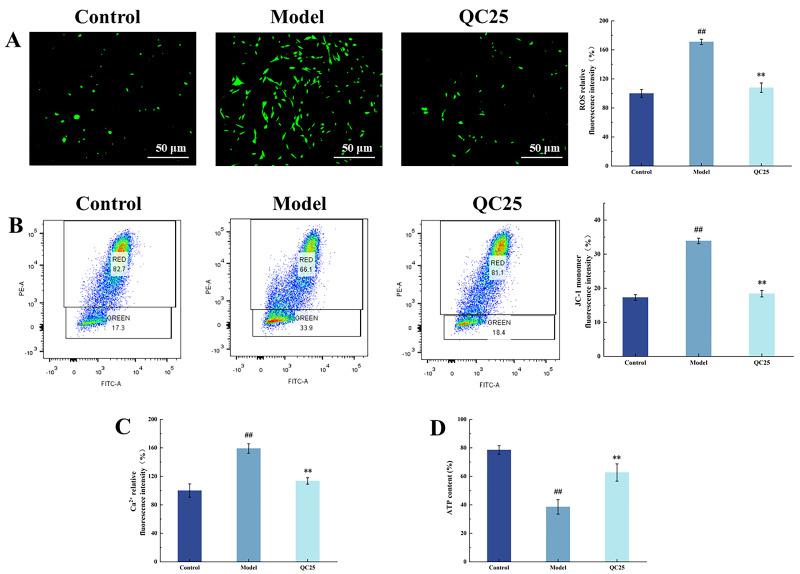
Effects of Different Broad Bean Extracts on Mitochondrial Function in Parkinson’s Disease Model Cells. (**A**) ROS Levels. (**B**) Mitochondrial Membrane Potential (Green Fluorescence Intensity). (**C**) Intracellular Calcium Ion Concentration. (**D**) ATP Content. Data are shown as the mean ± SEM, *n* = 5, ^##^ *p* < 0.01 versus Control group; ** *p* < 0.01 and versus Model group.

ROS serve as signaling molecules essential for diverse physiological processes. However, when physiological compensatory mechanisms fail to maintain ROS within homeostatic ranges, oxidative stress occurs, damaging mitochondrial function and promoting apoptosis [21]. Intracellular ROS levels were quantified using the DCFH-DA fluorescent probe, where green fluorescence intensity correlates positively with ROS production. Representative inverted fluorescence microscopy images and normalized ROS fluorescence intensities are shown in Figure 2A. In MPP^+^-treated PC12 cells, the fluorescence intensity reached 170% (^##^ *p* < 0.01), indicating the occurrence of oxidative stress. This is likely due to the release of ROS from Complex I into the mitochondrial matrix, and from Complex III into both the mitochondrial matrix and the inner membrane following MPP^+^ treatment, resulting in increased ROS generation. Notably, the QC25 extract significantly attenuated MPP^+^-induced overproduction of ROS (** *p* < 0.01), demonstrating its antioxidant protective effect on mitochondria.

Mitochondrial membrane potential directly reflects the integrity of the proton gradient across the inner mitochondrial membrane [22]. The fluorescent probe JC-1 was employed to assess ΔΨm, where depolarization reduces JC-1 aggregate formation and increases green fluorescence intensity. Figure 2B displays flow cytometry profiles and normalized JC-1 monomer fluorescence intensities across experimental groups. MPP^+^-treated cells exhibited a significant increase in green fluorescence intensity (^###^ *p* < 0.001), with JC-1 monomer proportion rising to 30.20%, indicating severe ΔΨm collapse. Mechanistically, MPP^+^ inhibits mitochondrial Complex I, disrupting electron transport chain activity and triggering ΔΨm dissipation [23]. QC25 treatment markedly attenuated these effects, reducing green fluorescence intensity and decreasing JC-1 monomer proportion to 14.16% (** *p* < 0.01), thereby restoring ΔΨm in damaged cells.

Ca^2+^ serve as pivotal signaling molecules in neuronal transmission and functional regulation, with their homeostatic balance being essential for maintaining normal neuronal activity [24]. When MPP^+^ inhibits mitochondrial Complex I, leading to collapse of the mitochondrial membrane potential (MMP), mitochondria lose their calcium-buffering capacity, resulting in a marked elevation of cytosolic Ca^2+^ concentrations [25]. As demonstrated in Figure 1C, MPP^+^-treated model cells exhibited a 160% increase in intracellular Ca^2+^ levels compared to the control group (^##^ *p* < 0.01), indicating severe disruption of calcium homeostasis. QC25 broad bean extract treatment significantly reduced Ca^2+^ overload (** *p* < 0.01), restoring the concentration to 103.55% of baseline. These findings demonstrate that QC25 alleviates mitochondrial calcium signaling burden, restores mitochondrial functionality, and mitigates apoptosis in Parkinson’s disease cell models.

ATP, primarily synthesized by the mitochondrial oxidative phosphorylation system, serves as a critical indicator of electron transport chain efficiency and cellular energy supply [26]. As shown in Figure 2D, MPP^+^ treatment induced a significant reduction in intracellular ATP levels compared to the control group (^##^ *p* < 0.01). Mechanistically, MPP^+^ selectively inhibits mitochondrial Complex I, disrupting electron transfer and proton gradient formation while triggering oxidative stress and mitochondrial membrane potential collapse, ultimately suppressing ATP synthesis [27]. Notably, QC25 broad bean extract treatment restored ATP content to 62.70% (** *p* < 0.01). These results suggest that QC25 may antagonize the toxic effects of MPP^+^ on mitochondrial energy metabolism by preserving Complex I functionality or enhancing oxidative phosphorylation efficiency

### 3.4. Effect of Broad Bean Extract on the PD Models Cell Metabolome by UPLC-QTOF/MS

#### 3.4.1. Metabolomic Profiling Analysis

**Figure 3:**
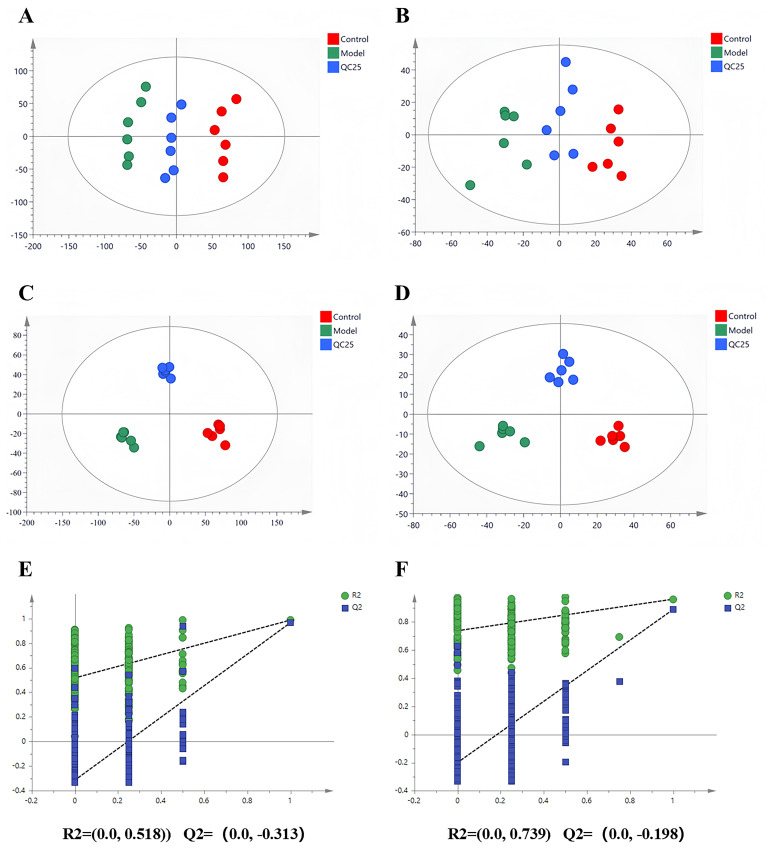
PCA scores of ESI+ (**A**) and ESI- (**B**) model in CON group, Model group and QC25 group. OPLS-DA scores of ESI+ (**C**) and ESI- (**D**) mode in CON group, Model group and QC25 group. Permutation test results of ESI+ (**E**) and ESI- (**F**) pattern in CON group, Model group and QC25 group.

Metabolomic data from the cell control, model (MPP^+^-treated), and QC25 groups were analyzed using multivariate statistical methods in SIMCA-P following UPLC-Q-TOF-MS acquisition. As shown in Figure 3A,B, principal component analysis (PCA) score plots revealed no outliers, with distinct clustering among groups. Notably, the control, model, and QC25 groups exhibited significant separation, and the trajectory of QC25-treated cells trended toward normalization.

Further validation via supervised partial least squares-discriminant analysis (PLS-DA) confirmed intergroup separation in both positive and negative ion modes Figure 3C,D, aligning with PCA results and confirming successful establishment of the MPP^+^-induced PD model and QC25 intervention efficacy. Model reliability was evidenced by Q^2^ regression lines intersecting the negative *y*-axis in permutation tests Figure 3E,F, demonstrating robust stability and predictive capacity without overfitting.

#### 3.4.2. Metabolic Pathway Analysis

By matching mass-to-charge ratios, molecular formulas, retention times, and secondary fragment ions against HMDB, MassBank, and PubChem databases, 66 potential differential metabolites were annotated among the control, model, and QC25-treated groups (Table S1).

**Figure 4:**
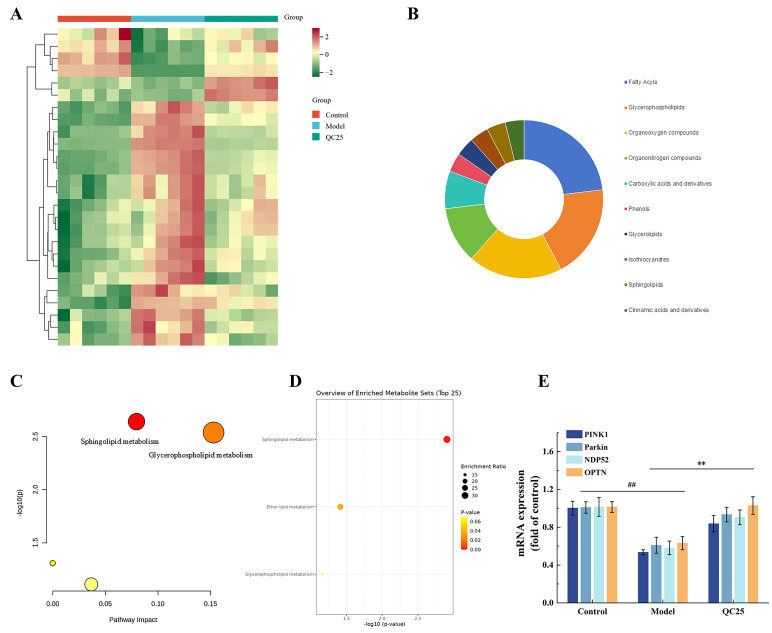
(**A**) Heatmap of different metabolites. (**B**) Classification of differential metabolites. (**C**) The KEGG enrichment analysis showed highly affected metabolites sets. (**D**) The KEGG analysis showed highly affected pathways. (**E**) The mRNA expression levels of PINK1, Parkin, NDP52, and OPTN in each group.Data are shown as the mean ± SEM, *n* = 5, ^##^ *p* < 0.01 versus Control group; ** *p* < 0.01 versus Model group.

As summarized in Table S1, MPP^+^ treatment significantly downregulated 7 metabolites and upregulated 19 metabolites in the model group compared to controls. A heatmap of the 26 differential metabolites (Figure 4A) visualized their dynamic changes across groups, with green-to-red gradients representing high-to-low relative abundances. Hierarchical clustering demonstrated stark metabolic disparities between the model and control groups, while the QC25-treated group exhibited intermediate profiles, suggesting partial reversal of MPP^+^-induced metabolic perturbations.

Figure 4B highlights that QC25 (broad bean extract) treatment predominantly downregulated metabolites in the MPP^+^-injured PC12 cell group. Specifically, the downregulated metabolites are mainly categorized into three key classes: Fatty Acyls (fatty acyls), Glycerophospholipids (glycerophospholipids), and Organooxygen compounds (organooxygen compounds)—which are closely associated with lipid metabolism homeostasis and oxidative stress regulation in neural cells. Pathway enrichment analysis via MetaboAnalyst 5.0 (Figure 4C) implicated two key pathways: glycerophospholipid metabolism and sphingolipid metabolism. Further functional enrichment (Figure 4D) revealed QC25-mediated reprogramming of Sphingolipid metabolism, Ether lipid metabolism, and Glycerophospholipid metabolism in PC12 cells.

These results suggest that the neuroprotective effects of broad bean extract may be mediated through regulation of the glycerophospholipid metabolism pathway in PC12 cells. It is well-established that glycerophospholipid metabolism is closely linked to mitochondrial functional stability [28]. The inner and outer mitochondrial membranes are predominantly composed of glycerophospholipids, whose composition and ratios directly determine membrane fluidity, stability, and functionality [29]. The extract may restore mitochondrial membrane architecture via modulation of glycerophospholipid metabolism.

The aforementioned metabolomics analysis indicated that the QC25 extract significantly reversed the MPP^+^-induced metabolic disturbances, particularly by regulating the glycerophospholipid metabolism pathway, which is closely associated with mitochondrial membrane structure and function. Given that mitochondrial dysfunction (such as collapse of membrane potential, ROS burst, and ATP depletion) serves as a key signal triggering mitophagy to eliminate damaged mitochondria, and considering that the PINK1/Parkin pathway represents the central mechanism regulating this process, we further investigated the effects of the QC25 extract on the PINK1/Parkin-mediated mitophagy pathway.

### 3.5. Effects of Broad Bean Extract on the PINK1/Parkin Pathway

As shown in Figure 4E, mRNA expression levels of PINK1 and Parkin in the model group were significantly reduced to 53.49% and 60.93%, respectively, compared to the control group (^##^ *p* < 0.01). This indicates that MPP^+^-induced inhibition of mitochondrial Complex I triggers sustained collapse of mitochondrial membrane potential (MMP), preventing PINK1 stabilization on the outer mitochondrial membrane and subsequent recruitment of Parkin to initiate mitophagy [30]. In contrast, QC25 treatment restored PINK1 and Parkin expression to 83.76% and 93.41%, respectively (** *p* < 0.01). These findings suggest that QC25 protects PD model cells by restoring PINK1/Parkin-mediated mitophagy.

NDP52 and OPTN, two critical autophagy receptors, facilitate lysosomal degradation of damaged mitochondria by interacting with ubiquitin tags on their surfaces [31]. The QC25 intervention significantly rescued the downregulated expression of NDP52 and OPTN (** *p* < 0.01), demonstrating its ability to enhance autophagic recognition of ubiquitinated mitochondria and improve mitophagic flux.

### 3.6. Screening of Levodopa-Synergizing Substances

**Figure 5:**
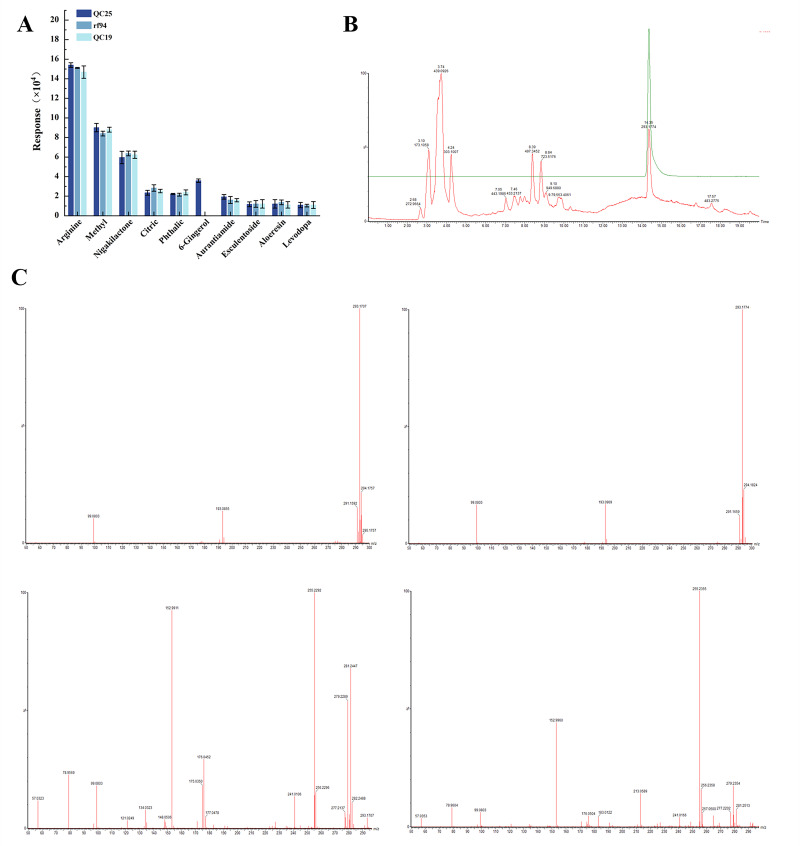
(**A**) UHPLC-Q/TOF-MS Identification of active ingredients in broad bean QC25, rf94 and QC15 (top ten substances with the highest response values). (**B**) Overlay of chromatograms of QC25 extract and 6-gingerol. The red chromatogram represents the sample, and the green chromatogram represents 6-gingerol. (**C**) MS and MS/MS spectra of QC25 extract and 6-gingerol. The spectra on the left correspond to 6-gingerol, and those on the right correspond to QC25 extract.

Based on the observed variations in cellular activity induced by different broad bean extracts under identical levodopa administration (Section 3.1), it was hypothesized that certain components within the extracts may potentiate the protective effects of levodopa. Therefore, three broad bean extracts (QC25, rf94, and QC19) were subjected to UHPLC-Q/TOF-MS analysis under identical levodopa concentrations. The top ten compounds ranked by intensity in the analysis results were summarized in Figure 5A, and information such as retention time and characteristic ion fragments for each compound was compiled in Table 3. As shown in Figure 5A, among the top 10 compounds ranked by response intensity, there were no significant differences between all substances except 6-gingerol. However, QC25 contained 6-gingerol, which was not detected in the other two varieties. Figure 5B shows the overlaid chromatograms of the extract and 6-gingerol standard, while Figure 5C presents their side-by-side MS and MS/MS spectra, collectively confirming the presence of 6-gingerol in QC25. Notably, 6-GIN exhibits potent antioxidant properties, effectively neutralizing free radicals, alleviating oxidative stress, and protecting neuronal cells, potentially delaying neurodegeneration in Parkinson’s disease and Alzheimer’s disease [32]. This suggests that 6-GIN may account for the superior efficacy of QC25 compared to the other varieties in the Parkinson’s disease model cells.

### 3.7. Synergistic Effects of 6-Gingerol and Levodopa in Parkinson’s Model Cells

**Figure 6:**
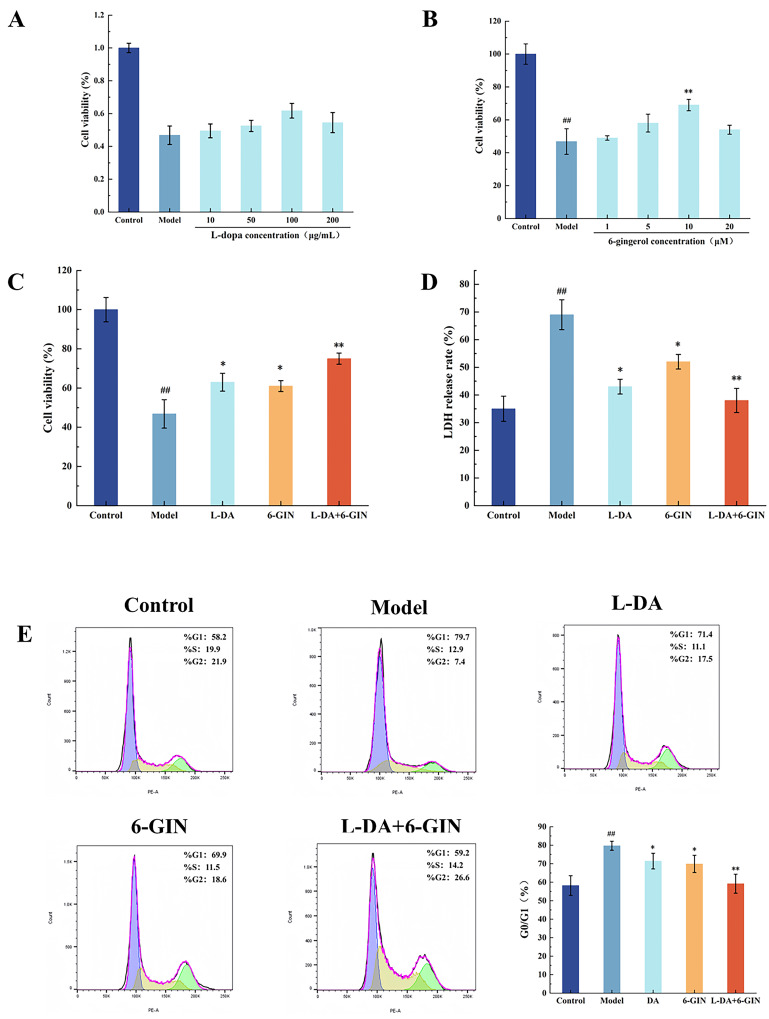
Effects of L-DA, 6-GIN, and L-DA + 6-GIN treatments on Parkinson’s disease model cells. (**A**) Screening for the Optimal Concentration of Levodopa. (**B**) Screening for the Optimal Concentration of 6-Gingerol. (**C**) Cell Viability. (**D**) Cell LDH Release Rate. (**E**) Cell Cycle. Data are shown as the mean ± SEM, *n* = 5, ^##^ *p* < 0.01 versus Control group; * *p* < 0.05, ** *p* < 0.01 versus Model group.

As shown in Figure 6C, cell viability in all treatment groups was significantly higher than in the model group. The combination therapy group exhibited the strongest protective effect, with viability increasing to 75% (** *p* < 0.01). Correspondingly, LDH release rates in the treatment groups (Figure 6D) were significantly reduced to 38% (** *p* < 0.01), with the combination group showing a significant improvement compared to monotherapy groups. Similar results were observed in the cell cycle analysis (Figure 6E): The proportion of cells in the G0/G1 phase declined to 60% in the synergistic treatment group (** *p* < 0.01). These results confirm that co-administration of L-DOPA and 6-GIN synergistically maintains cell membrane integrity, alleviates MPP^+^-induced cytotoxicity, and enhances protection against Parkinson’s disease-associated cellular damage.

### 3.8. Synergistic Effects of 6-Gingerol and Levodopa on Mitochondrial Function

**Figure 7:**
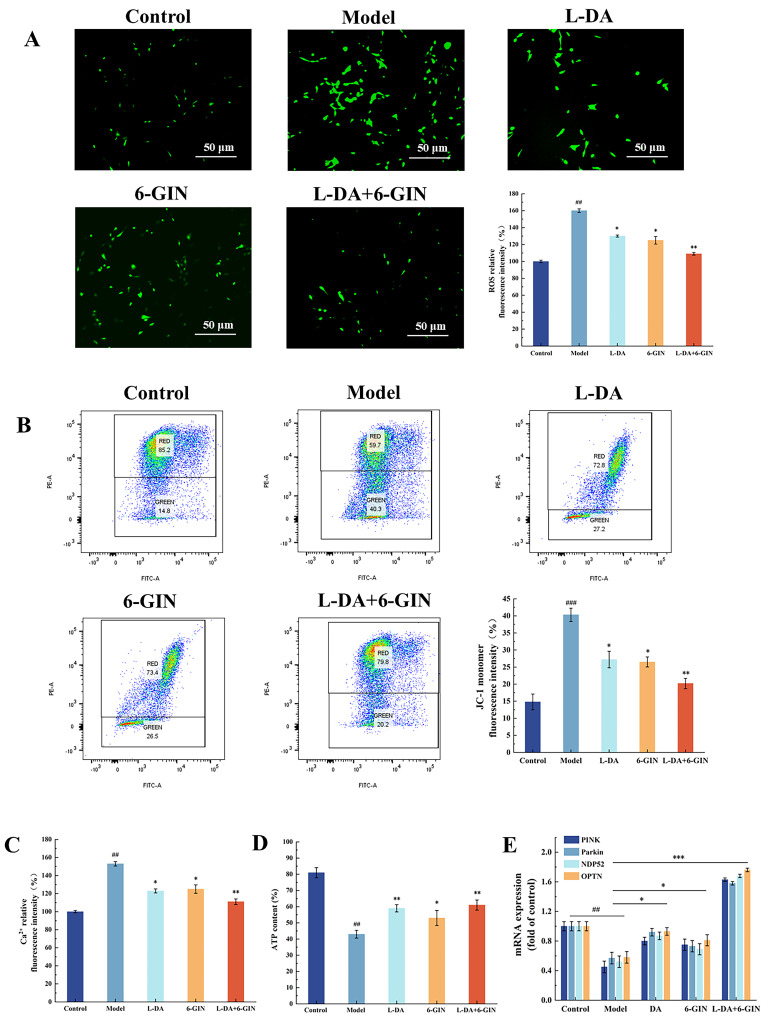
(**A**) Effects of L-DA, 6-GIN, and L-DA+6-GIN Treatment on Intracellular ROS Levels. (**B**) Mitochondrial Membrane Potential. (**C**) Calcium Ion Concentration. (**D**) ATP Content. (**E**) The mRNA expression levels of PINK1, Parkin, NDP52, and OPTN in each group. Data are shown as the mean ± SEM, *n* = 5, ^##^ *p* < 0.01 and ^###^ *p* < 0.001 versus Control group; * *p* < 0.05, ** *p* < 0.01 and *** *p* < 0.001 versus versus Model group.

As shown in Figure 7A,B, both 6-GIN and levodopa significantly reduced ROS release and the green fluorescence intensity of mitochondrial membrane potential, with the combined treatment exhibiting a synergistic effect (** *p* < 0.01). These results indicate that the co-treatment can synergistically alleviate oxidative stress and regulate mitochondrial membrane potential in PC12 cells. Figure 7C,D further shows that the co-treatment can synergistically restore the calcium ion content and ATP levels in PC12 cells (** *p* < 0.01). The combined treatment not only effectively inhibits MPTP opening and reduces calcium efflux into the cytoplasm but also restores cellular energy supply, supporting the repair of mitochondrial function. In summary, these findings demonstrate that the combined medication can not only improve oxidative stress and ATP synthesis but also directly stabilize MMP, addressing mitochondrial dysfunction in the PD model. This synergistic effect may stem from complementary mechanisms of cellular energy metabolism regulation and mitochondrial function enhancement, which jointly promote energy recovery and mitochondrial protection.

### 3.9. Regulation of the PINK1/Parkin Pathway by 6-Gingerol and Levodopa Combination

In the model group, PINK1 and Parkin mRNA expression decreased to 45.21% and 57.37% of control levels, respectively (^##^ *p* < 0.01; Figure 7E). Levodopa monotherapy showed weaker restoration of PINK1/Parkin expression compared to 6-GIN, likely because levodopa indirectly mitigates mitochondrial damage by improving dopaminergic neuronal metabolism and alleviating energy deficits [33]. In contrast, 6-GIN directly scavenges mitochondrial ROS, stabilizes MMP, and promotes PINK1 accumulation on the outer mitochondrial membrane [34]. The combination therapy synergistically restored PINK1 and Parkin expression to 163.02% and 158.07% (*** *p* < 0.001), respectively, through dual mechanisms: rescuing cellular energy homeostasis and repairing mitochondrial integrity.

Compared to the model group, co-treatment with levodopa and 6-GIN significantly upregulated NDP52 and OPTN expression to 168.17% and 176.05%, respectively (*** *p* < 0.001). These findings suggest that the combined treatment can enhance mitophagy by promoting autophagic receptor recognition of ubiquitinated mitochondria, accelerating autophagosome formation, and facilitating subsequent lysosomal degradation.

## 4. Conclusions

This study identifies QC25, a broad bean cultivar rich in L-DOPA, phenolics, and flavonoids, as a promising functional food ingredient for supporting mitochondrial health and neuronal resilience. QC25 alleviates oxidative stress, restores energy metabolism, and stabilizes mitochondrial membrane integrity in Parkinson’s disease models, while its bioactive component 6-gingerol synergizes with plant-derived L-DOPA to enhance mitophagy via PINK1/Parkin pathway activation. These findings highlight the potential of QC25 in functional foods to promote cellular antioxidant defenses and mitochondrial quality control, offering a dietary strategy for maintaining neurological health in aging populations. Further research should focus on optimizing QC25-based formulations for practical nutritional applications.

## Figures and Tables

**Table 1:** Sequences of the forward and reverse primers used in the qRT-PCR assay.

Gene	Forward Primer (5′→3′)	Reverse Primer (5′→3′)
*PINK1*	TGACCCACTGGACACACGAC	GCTCCCTTTGAGACGACATC
*PARKIN*	ACGCTCAACTTGGCTACTCC	CACTCCTCGGCACCATACTG
*OPTN*	GGACAGCCCTTGTGAGACCC	TCAAATCGCCCTTTCATAGC
*NDP52*	CGCTACCAGTTCTGCTATGTGGA	TCATGGAGGTCAGCGTACTTGTC
*GAPDH*	GACATGCCGCCTGGAGAAAC	AGCCCAGGATGCCCTTTAGT

**Table 2:** Results of determination of active components in broad bean extraction.

	Content	QC25	4030	rf94	QC14	QC15	QC16	QC18	QC19	QC21	lf18
Breed											
Levodopa		14.89 ± 0.87%	10.01 ± 0.82%	15.24 ± 1.42%	10.53 ± 0.60%	5.59 ± 0.53%	6.38 ± 1.78%	9.94 ± 0.44%	8.47 ± 0.17%	9.7 ± 0.80%	5.33 ± 0.08%
Total phenol (mg GAE/g)		27.52 ± 0.20%	19.64 ± 0.28%	13.71 ± 0.23%	11.42 ± 0.08%	6.44 ± 0.19%	5.50 ± 0.06%	10.18 ± 0.09%	8.93 ± 0.07%	13.16 ± 0.09%	8.69 ± 0.14%
Total flavone (mg RE/g)		3.32 ± 0.08%	1.45 ± 0.03%	2.78 ± 0.04%	1.22 ± 0.03%	1.55 ± 0.03%	1.65 ± 0.04%	0.73 ± 0.03%	0.84 ± 0.04%	0.92 ± 0.05%	0.73 ± 0.02%

**Table 3:** Identification data of the top ten compounds in QC25 obtained by UPLC-QTOF/MS analysis.

Mode	No.	Component	RT	Observed *m*/*z*	Molecular Formulas	Fragment Ions (*m*/*z*)
ESI+	1	Arginine	0.95	175.1178	C6H14N4O2	70.0664, 1052.0
ESI-	2	Methyl lucidenate Q	19.06	473.2906	C28H42O6	473.29012, 208.00186
ESI+	3	Nigakilactone H	15.84	425.2147	C22H32O8	129.01709, 365.19426
ESI-	4	Citric acid_1	2.15	191.0211	C6H8O7	111.1, 100.0
ESI+	5	Phthalic anhydride	14.78	149.022	C9H6O3	163.038971, 99.999999
ESI-	6	6-Gingerol	11.23	293.1797	C17H26O4	99.0815, 193.090600
ESI-	7	Aurantiamide acetate	4.04	443.2005	C27H28N2O4	194.1176, 21818032.0
ESI+	8	Esculentoside A	15.84	827.4432	C24H66O16	425.21474, 129.01709
ESI-	9	Aloeresin C	1.41	701.2056	C34H38O16	383.12500, 89.02377
ESI+	10	Levodopa	2.39	198.075	C9H11NO4	152.0, 64632.0

## Data Availability

The original contributions presented in this study are included in the article/Supplementary Material. Further inquiries can be directed to the corresponding author.

## Supplementary Materials

The following supporting information can be downloaded at: https://www.mdpi.com/article/10.3390/foods14183244/s1, Table S1. MPP+-induced differential metabolites of mitochondrial damage in Parkinson’s cell models. (* *p* < 0.05, ** *p* < 0.01, *** *p* < 0.001; ^#^ *p* < 0.05, ^##^ *p* < 0.01, ^###^ *p* < 0.001).
