# Supplementary material for: Protective Mechanism of Broad Bean Extract on Parkinson’s Disease Model Cells
Source: Foods. 2025 Sep 18;14(18):3244. doi: 10.3390/foods14183244 (PMC12469533; doi:10.3390/foods14183244)
Supplement: Supplementary file 1 [file foods-14-03244-s001.zip › foods-3805393-supplementary.pdf]

Table S1. MPP+-induced differential metabolites of mitochondrial damage in Parkinson's cell models. (* $p < 0.05$, ** $p < 0.01$, *** $p < 0.001$; $^{\#}$ $p < 0.05$, $^{\#\#}$ $p < 0.01$, $^{\#\#\#}$ $p < 0.001$).

Mode	No.	Name	HMDBID	Formula	vip	*p* Value	MPP^+/ CON	QC25/ MPP^+
ESI+	1	Hydroxytyrosol 1-O-glucoside	HMDB0041024	$C_{14}H_{20}O_8$	2.14216	0.006850125	↑**	↓##
ESI+	2	3-(3,4-Dihydroxyphenyl)-1-propanol 3'-glucoside	HMDB0038304	$C_{15}H_{22}O_8$	1.80356	0.032631554	↑*	↓#
ESI+	3	threo-Syringoylglycerol	HMDB0031237	$C_{11}H_{16}O_6$	2.01539	0.004179121	↑**	↓##
ESI+	4	2-hexadecenal	HMDB0060482	$C_{16}H_{30}O$	1.37513	5.77991E-05	↑***	↓###
ESI+	5	3-Palmitoyl-sn-glycerol	HMDB0245964	$C_{19}H_{38}O_4$	1.20957	4.81662E-05	↑***	↓###
ESI+	6	PG(18:2(9Z,12Z)/22:5(4Z,7Z,10Z,13Z,16Z))	HMDB10657	$C_{46}H_{77}O_{10}P$	1.34336	6.02858E-07	↑***	↓###
ESI+	7	Oleamide	HMDB0002117	$C_{18}H_{35}NO$	2.54944	0.000177887	↑***	↓###
ESI+	8	PA(20:5(5Z,8Z,11Z,14Z,17Z)/22:1(13Z))	HMDB0115216	$C_{45}H_{77}O_8P$	1.01947	0.000137987	↑***	↓###
ESI+	9	Docosanamide	HMDB0000583	$C_{22}H_{45}NO$	1.78317	1.95519E-05	↑***	↓###
ESI+	10	N-stearoyl valine	HMDB0241952	$C_{23}H_{45}NO_3$	1.27716	1.65379E-05	↑***	↓###
ESI+	11	dexpanthenol	HMDB0004231	$C_9H_{19}NO_4$	1.37943	0.000403337	↑***	↓###
ESI+	12	Stearamide	HMDB0034146	$C_{18}H_{37}NO$	12.9289	1.19265E-05	↑***	↓###
ESI+	13	Phytosphingosine	HMDB0004610	$C_{18}H_{39}NO_3$	8.23078	1.26409E-10	↑***	↓###
ESI+	14	Sphinganine	HMDB0000269	$C_{18}H_{39}NO_2$	2.18238	3.77886E-10	↑***	↓###
ESI+	15	Galabiose	HMDB0029902	$C_{24}H_{44}O_{22}$	1.1482	4.10111E-07	↓###	↑***
ESI+	16	Cer(d18:0/12:0)	HMDB0011758	$C_{30}H_{61}NO_3$	1.46714	4.94049E-14	↑***	↓###
ESI+	17	Palmitic amide	HMDB0012273	$C_{16}H_{33}NO$	3.00489	0.003433275	↑**	↓##
ESI+	18	Arachidoyl Ethanolamide	HMDB0248559	$C_{22}H_{45}NO_2$	1.22794	3.06059E-07	↑***	↓###
ESI+	19	N-acetylsphinganine	HMDB0249513	$C_{20}H_{41}NO_3$	1.69803	0.000181425	↑***	↓###
ESI+	20	Dodeca-7,9-dienedioylcarnitine	HMDB0241256	$C_{24}H_{36}O_3$	1.08152	9.17304E-10	↓###	↑***
ESI+	21	Glycerophosphocholine	HMDB0000086	$C_8H_{20}NO_6P$	1.30235	6.16076E-11	↓###	↑***
ESI-	22	p-Coumaroylagmatine	HMDB0033460	$C_{14}H_{20}N_4O_2$	1.38916	1.17874E-06	↓###	↑***
ESI-	23	PA(18:0/20:4(5Z,8Z,11Z,14Z))	HMDB0114884	$C_{41}H_{73}O_8P$	1.30862	4.56404E-07	↓###	↑***
ESI-	24	PC(15:0/0:0)	HMDB0010375	$C_{23}H_{48}NO_7P$	1.80462	2.14828E-13	↑***	↓###
ESI-	25	gamma-Glutamyl-beta-cyanoalanine	HMDB60478	$C_9H_{13}N_3O_5$	1.87497	2.03822E-08	↓###	↑***
ESI-	26	Sebacic acid	HMDB0000792	$C_{10}H_{18}O_4$	1.27786	0.007670156	↓##	↑**
